# Pyogenic Odontoid Osteomyelitis with Sinus Thrombosis

**DOI:** 10.1155/2017/2507645

**Published:** 2017-10-29

**Authors:** Rohit Aiyer, Janice Hwang, Edward H. Yu

**Affiliations:** ^1^Department of Psychiatry, Hofstra Northwell Health, Staten Island University Hospital, Staten Island, NY, USA; ^2^Department of Radiology, Hofstra Northwell Health, Staten Island University Hospital, Staten Island, NY, USA; ^3^Department of Neurology, Hofstra Northwell Health, Staten Island University Hospital, Staten Island, NY, USA

## Abstract

71/F presented with left sided headaches and neck pain with nuchal rigidity progressively worsening over 3 weeks with no other neurologic symptoms. Odontoid osteomyelitis with epidural abscess was discovered on further workup with neuroimaging. Concurrent jugular vein and transverse sinus venous thrombosis was also found and suspected to be secondary to the pyogenic odontoid osteomyelitis. Patient was treated with intravenous antibiotics for the osteomyelitis as well as intravenous heparin for the venous thrombosis. To our knowledge, this is the first case reported in literature of transverse sinus venous thrombosis secondary to odontoid osteomyelitis.

## 1. Introduction

Cervical vertebral osteomyelitis readily responds to conservative treatments if detected early but can lead to significant morbidity if fulminant due to adjacent infectious and inflammatory changes. Osteomyelitis affecting the cranium can lead to thrombosis of the subjacent cerebral venous sinus, often seen in untreated mastoiditis resulting in lateral or transverse sinus thrombosis [1]. Extracranial thrombosis of the internal jugular vein can result from adjacent oropharyngeal abscesses resulting in Lemierre's syndrome. We describe a patient presenting with spontaneous cervical osteomyelitis affecting the odontoid process with thrombosis of the adjacent internal jugular vein extending intracranially into cerebral sinuses.

## 2. Case Report

We present a 71/F presenting with a left occipital headache and neck pain progressing over 3 weeks. Pain was persistent and radiated down the left side of her neck. Vital signs were normal and physical examination was significant for nuchal rigidity with reproducible pain on head movement and limited range of motion. Cervical paraspinal muscles were tender to palpation without Spurling sign. There were no other neurologic deficits noted. Serologic testing revealed lymphocyte predominant leukocytosis (total WBC 16,500/mm^3^; lymphocyte 1,110/mm^3^, monocyte 350/mm^3^, granulocyte 14,570/mm^3^, eosinophils 10/mm^3^, and basophils 20 mm^3^). Inflammatory markers were elevated with erythrocyte sedimentation rate 120 mm/hr and c-reactive protein of 13.95 mg/dl.

**Figure 1:**
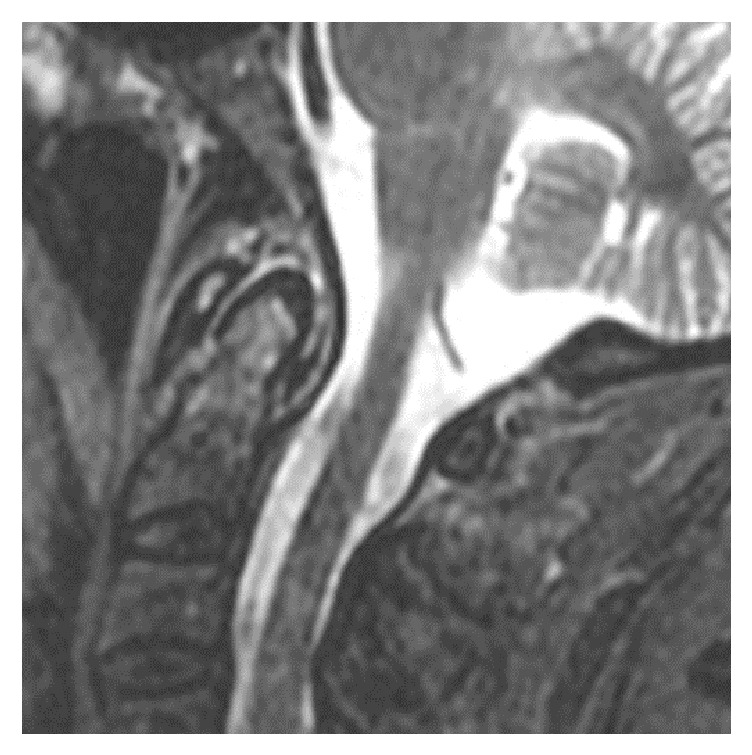
Sagittal STIR sequence demonstrating bone marrow edema within the C2 odontoid process. There are also high signal changes in the surrounding soft tissues reflecting related inflammation.

**Figure 2:**
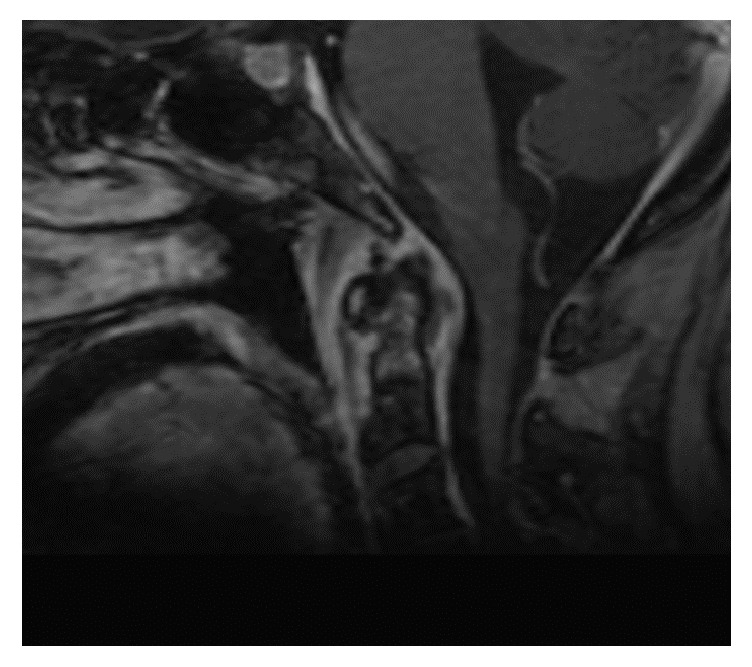
Sagittal postcontrast sequence demonstrating enhancement within the C2 odontoid process and surrounding prevertebral and epidural enhancing inflammatory soft tissue with liquefaction.

**Figure 3:**
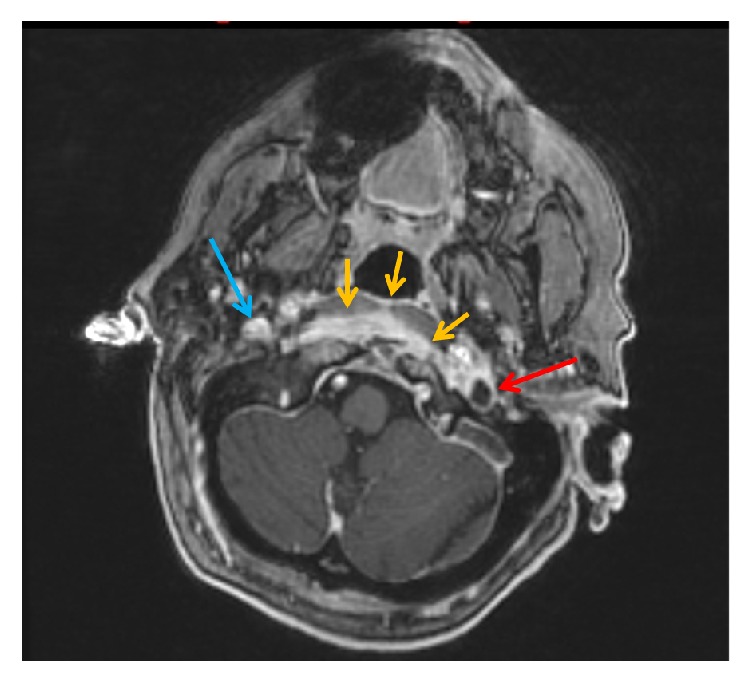
Postcontrast axial FSPGR sequence demonstrating filling defect within the left sigmoid sinus and proximal jugular vein (red arrows). Compare with the normal appearing right jugular vein (blue arrow). Also seen is inflammatory soft tissue in the prevertebral space (orange arrows) which extends into the epidural space behind the dens.

**Figure 4:**
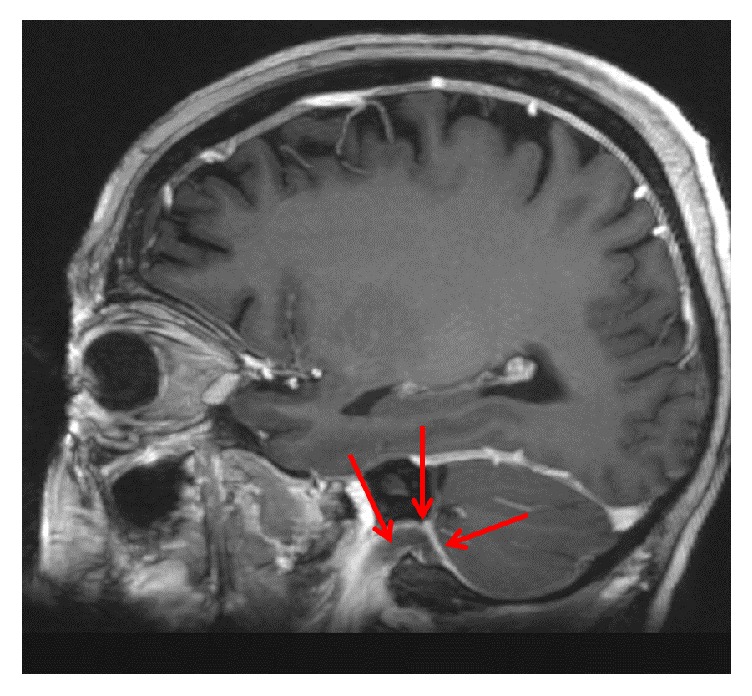
Sagittal reformatted postcontrast FSPGR images demonstrating filling defect within the left sigmoid sinus and proximal jugular vein. Arrow demonstrates thrombus within the distal sigmoid sinus and internal jugular vein.

**Figure 5:**
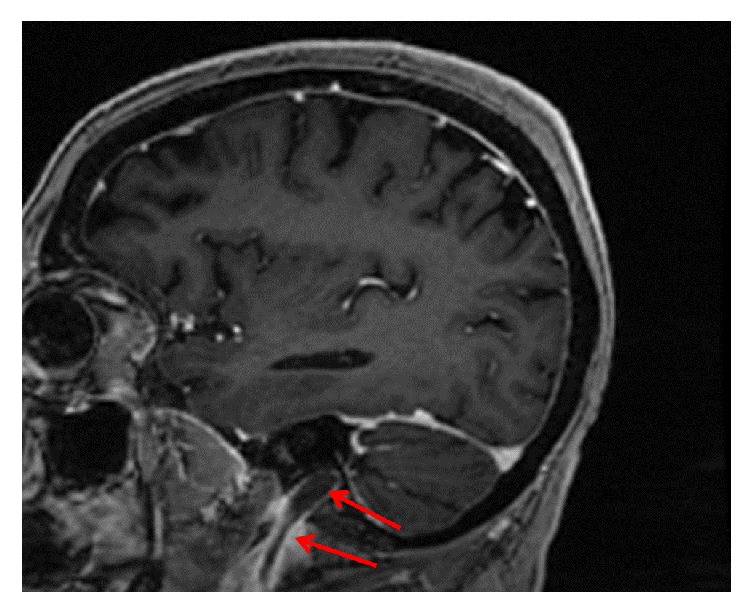
Sagittal reformatted postcontrast FSPGR images demonstrating filling defect within the left sigmoid sinus and a clearer view of the jugular vein. Arrow demonstrates thrombus within the distal sigmoid sinus and internal jugular vein.

**Figure 6:**
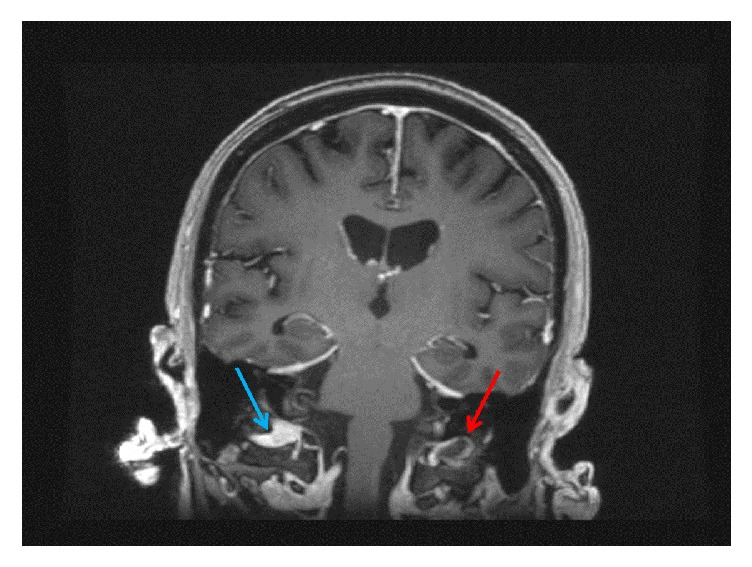
Coronal reformatted postcontrast FSPGR sequence demonstrating filling defect within the left sigmoid sinus. Blue arrow is demonstrating a normal right vein. Red arrow is demonstrating clot within the left vein.

**Figure 7:**
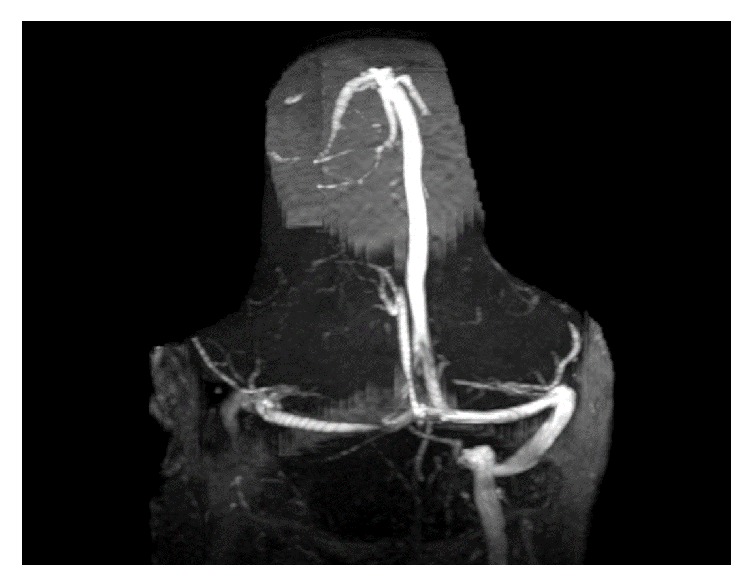
Time of flight MRV image demonstrating absence of signal within the left sigmoid sinus and proximal jugular vein.

MRI of the cervical spine revealed bone marrow edema within the C2 odontoid process with surrounding prevertebral and ventral epidural inflammation with an area of liquefaction posterior to the dens (Figures 1–3) suggestive of an infectious process. Magnetic resonance imaging (MRI, General Electric 3 Tesla) of the brain with and without gadolinium (DTPA) contrast revealed a near occlusive filling defect of the left sigmoid sinus extending into the left jugular vein (Figures 4–6). Enhancement of the prevertebral and ventral epidural space was suspicious for an epidural abscess. Magnetic resonance venography (MRV) revealed soft tissue signal with decreased flow suggestive of a thrombosis within the left sigmoid sinus and internal jugular vein (Figure 7). Echocardiogram and serial blood cultures were negative.

The patient was anticoagulated with intravenous unfractionated heparin with eventual conversion to long term oral anticoagulation with warfarin. Broad spectrum antibiotics were initiated and continued for 6 weeks. After 6 weeks of treatment, our patient had near complete resolution of cervical pain and headache with no significant neurologic deficits. Blood cultures remained negative throughout hospitalization.

## 3. Discussion

Vertebral osteomyelitis is less commonly associated with the cervical spine than with thoracic or lumbar segments accounting for only 12% of spinal osteomyelitis cases and is particularly rare affecting the odontoid process [2]. Cervical osteomyelitis occurs in 0.2–1.2 patients per 10,000 hospital admissions [3] with few cases involving the odontoid process specifically. Rheumatic destruction of the odontoid process is more common with the predominance of articular surfaces within the atlantoaxial articulation, which is distinct from other spinal cord segments [2]. While computed tomography (CT) is useful for locating areas of cortical bone destruction, prevertebral swelling, and delineation of abscesses [4], MRI remains the definitive study for early diagnosis of vertebral osteomyelitis with sensitivity of 95% and specificity of 90% [5]. T1 and T2 weighted sequences can also be used to differentiate complicated from uncomplicated osteomyelitis by examining involvement of the surrounding soft tissue.

Pathogenesis for vertebral osteomyelitis is usually due to local invasion of infectious organisms with progression to abscess formation through accumulation of pus in adjacent soft tissue spaces. Pyogenic osteomyelitis usually occurs by hematogenous spread in the absence of open trauma. Incidence of associated comorbid conditions includes diabetes mellitus in 24% of patients, followed by cancer (16%), intravenous drug users (10%), and corticosteroid treatment (7%) [6]. The most common pathogen is* Staphylococcus aureus*, which accounts for 40–80% of all cases [7].

Cerebral venous thrombosis (CVT) has an annual incidence of 1 per 100,000 patients [8]. Risk factors for CVT include prothrombotic conditions, infections, and malignancy. More than one risk factor increases the risk of CVT with over half of all CVT patients having 2 or more risks factors and 13% with no risks [9]. Intracranial infections as a cause for CVT are relatively rare accounting for only 6–12% of all CVTs [9]. Sinus venous thrombosis associated with vertebral osteomyelitis is even more rare with scant literature showing correlation.

Vertebral osteomyelitis with CVT is uncommon likely due to anatomic factors since most infections are detected before extension into nonadjacent vertebral bodies or skull base. Neurologic complications from osteomyelitis often arise from epidural abscess formation and since the dura is tightly adherent to the foramen magnum separating intracranial dural space from the spinal thecal sac, epidural abscesses typically do not cross from thecal sac intracranially. Infection can spread through hematogenous means since both arterial and venous systems span both intracranial and spinal segments. Vertebral osteomyelitis in high cervical segments such as the odontoid process can spread to adjacent soft tissue resulting in adherence of fibrin, blood, and platelets producing thrombosis which can extend intracranially into the sigmoid and transverse sinus as in our patient.

Mortality and dependency rates of CVT remain high with an estimated 15% of cases resulting in death or significant disability [9]. Complications occur in the form of venous infarcts and early detection leads to more favorable outcomes with early initiation of anticoagulation to prevent progression of thrombosis with resultant venous infarcts. Treatment for osteomyelitis requires long term intravenous antibiotics and possible surgical excision of epidural abscesses or empyema if present. Osteomyelitis affecting the odontoid process is rare and special consideration should be made when treating these infections since it is well situated to cause cerebral venous thrombosis due to continuity between of the internal jugular vein with cerebral venous sinuses. Since these two entities require very different treatment approaches, CVT as a potential cause for neurologic complication should be assessed in any patient with high cervical infections.
